# Research to Improve Fixed Orthodontic Treatment of Thirty Six Angle Class II Severe Malocclusions with Premolar Extractions Using a Modified Orthodontic Scientific Simulator

**DOI:** 10.3390/bioengineering13010041

**Published:** 2025-12-29

**Authors:** Radu Mircea Pisc, Anne-Marie Rauten, Mihai Raul Popescu, Mihaela Ionescu, Oana Gîngu, Stelian-Mihai-Sever Petrescu, Horia Octavian Manolea

**Affiliations:** 1 Department of Orthodontics, Faculty of Dental Medicine, University of Medicine and Pharmacy of Craiova, 200349 Craiova, Romania; radu.pisc@gmail.com (R.M.P.); rautenannemarie@yahoo.com (A.-M.R.); 2 Department of Occlusology and Fixed Prosthetics, Faculty of Dental Medicine, University of Medicine and Pharmacy of Craiova, 200349 Craiova, Romania; popescumihairaul@yahoo.com; 3 Department of Medical Informatics and Biostatistics, Faculty of Dental Medicine, University of Medicine and Pharmacy of Craiova, 200349 Craiova, Romania; mihaela.ionescu@umfcv.ro; 4 Department of Automotive, Transportation and Industrial Engineering, Faculty of Mechanics, University of Craiova, 200478 Craiova, Romania; oana.gingu@edu.ucv.ro; 5 Department of Dental Materials, Faculty of Dental Medicine, University of Medicine and Pharmacy of Craiova, 200349 Craiova, Romania; horia.manolea@umfcv.ro

**Keywords:** angle class II malocclusion, electrodont, fixed orthodontic treatment, orthodontic scientific simulator, TiNb wires

## Abstract

This study aimed to evaluate the new orthodontic TiNb wires in direct comparison to the gold standard in orthodontics, NiTi wires, when treating. There is limited literature on patients with severe malocclusions being treated from start to end with TiNb, and TiNb wires were mostly used in the final stages of treatment. Our protocol consisted of three orthodontic wires: 0.016, 0.016 × 0.025, and 0.019 × 0.025 for levelling and aligning the stage and 0.019 × 0.025 stainless steel for the finishing stage, in order to treat the same case reproduced on a modified scientific simulator. The bracket system used was made by GC slot 0.22, TiNb wires made by Morita, and NiTi wires produced by GC. We ligated all brackets using SS wire ligatures 0.008, and for anchorage, we used a transpalatal arch. The temperature of the scientific simulator was set between 20 and 25 degrees Celsius. We have used upper arches and studied the repositioning of upper ectopic canines and space closure in order to obtain an equilibrated maxillary arch. After each change of orthodontic wires, we scanned the upper arch using Medit i600 (Medit, Seoul, Republic of Korea). After concluding all stages on all upper arches, we assessed the results using LITTLE’s Irregularity index and stereo microscopy to explain metal stress on NiTi and TiNb. We propose an optimized process for using TiNb and NiTi wires when treating class II severe malocclusions with premolar extractions. Thus, we observed permanent deformation for all 0.016 TiNb wires used in the first stage, so TiNb underperformed in comparison with NiTi. Also, the Little’s Irregularity Index was superior in the NiTi wires group on 0.016 wires, verifying the change of state in the TiNb wires group.

## 1. Introduction

In daily practice, we observe an increased percentage of complicated cases with severe malocclusions and ectopic canines as a result of a lack of space for natural alignment. Fixed orthodontic devices are among our treatment choices, and we put to the test the new orthodontic TiNb archwires and the golden standard in orthodontics, NiTi archwires, when treating a Class II severe malocclusion with premolar extraction using the Strait Wire method and a transpalatal arch [1]. This type of treatment not only addresses cases with transversal or sagittal discrepancies but also addresses the vertical dimension (ectopic and high-positioned canines) and closes spaces after extractions [2]. During the space-closing stage, we produce bodily movements of dental units, and we need special characteristics of wires (rigid and with a low frictional profile) to finish the case due to frictional forces [3].

After researching the available literature regarding the new TiNb properties and finding the right moment to use it in treating a severe malocclusion with extractions, the information was intriguing because there was little to no information about patients with severe malocclusion being treated with TiNb. The TiNb wires were mostly used in the final stages of treatment [4,5]. Nitinol, developed in the early 1960s, was introduced to the field of orthodontics in the late 1970s [6]. Nickel-titanium (NiTi) wires are extensively utilized in orthodontics. These wires have streamlined the initial stages of orthodontic treatment due to their ability to exert low forces over a broad range of activation and their superelastic properties [7]. Nonetheless, the limited deformability of NiTi archwires restricts their application in the concluding stages of orthodontic therapy.

Gummetal is a novel multifunctional β-Ti alloy composed of titanium, niobium, tantalum, zirconium, and oxygen (TiNbTaZrO), which renders it bioinert [8,9,10]. Developed in 2001 by the Metallurgy Research Section of Toyota Central R&D, Inc. in Japan [11], Gummetal’s chemical composition (Ti-23Nb-0.7Ta-2Zr-1.2O) is based on atomic valence theory. The alloy undergoes intensive cold-working to achieve its unique properties. According to Hasegawa, Gummetal wire can reduce friction between the archwire and metal brackets by up to 50% compared to other titanium wires [12]. It features a remarkably low Young’s Modulus that remains constant with temperature and possesses high tensile strength, which is exceptionally rare, while exerting lower force than NiTi and β-Ti archwires. Manufacturers claim that the dislocation motion of the crystal (plastic deformation) is entirely controlled, distinguishing Gummetal from others [13].

The new approach in modern oral medicine, and in particular orthodontics, is to limit the use of materials, such as nickel, in daily practice due to its allergenic profile; the most common immunological reaction is contact hypersensibility when placed in the patient’s mouth. As orthodontists, we appreciate the versatility of a wire to be used from the initial to the final stages [14,15].

The paper submitted for publication refers to innovative research to improve the use of orthodontic arches in orthodontic offices. It highlights the limitations that arise when using TiNb arches at the beginning of treatment and offers solutions for carrying out the treatment offered to allergic patients or those for whom the use of the TiNb variant was chosen.

This study aims to offer insight into orthodontic fixed treatment focused on time in treatment when addressing class II malocclusion patients with severe crowding and premolar extractions, using TiNb as a new solution to the gold standard of NiTi wires.

## 2. Materials and Methods

The present study was approved by the Ethics Committee of the University of Medicine and Pharmacy of Craiova, Romania (approval reference no. 56/29 January 2024), in accordance with the ethical guidelines of the University of Medicine and Pharmacy of Craiova, Romania.

This study was conducted on a modified orthodontic scientific simulator. The advantage of this method is given by the preservation of the general characteristics of the two arches, modifying only the parameters of the ectopic canines analyzed. The simulated cases were made by increasing by 1 mm in relation to the two planes studied for each of the simulated cases, obtaining a representation from low to severe regarding the degree of canine ectopy. The study sample was represented by the 36 cases of severe malocclusions with ectopic canines and extractions of first premolars, obtained from these simulations performed up to a maximum deviation of 6 mm in each plane.

**Figure 1:**
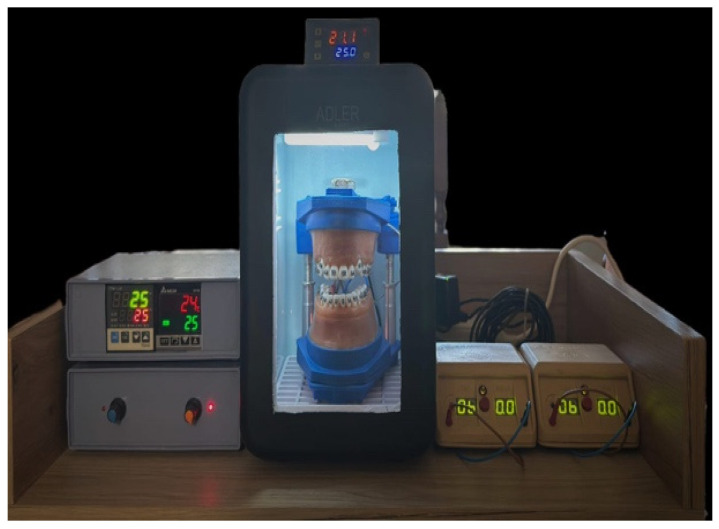
Scientific orthodontic simulator with temperature control and thermo controlled chamber.

We selected to treat in vitro orthodontically these thirty-six cases with different degrees of angulation and height of the canine in vertical and transversal planes reported to the occlusal plane, on a modified orthodontic scientific simulator that includes an electodont made by Savaria-Dent Kft (Szombatheley, Hungary) inside a controlled environment with Peltier effect for heating or cooling, as shown in Figure 1.

Additionally, we incorporated an amperage controller for each dental arch and embedded thermo sensors in wax to monitor its temperature, ensuring it remains stable between 20 and 25 degrees Celsius during the active phase [16]. When the wax temperature hit 25 degrees, the thermo sensor would cut off the current to the electrodont, allowing the controlled environment, or thermo chamber, to maintain a temperature of 24–25 degrees Celsius. This cycle continued automatically until the orthodontic wires used in treatment transferred all necessary information to the electrodont dental units, facilitating leveling and alignment. The cases involved severe crowding, ectopic canines, and premolar extractions to create space for the canines. The equipment used is a TTM-J4-R-AB digital temperature controller manufactured by TOHO ELECTRONICS INC. (Sagamihara, Japan). The temperature controller receives information about the actual temperature of the process and compares it with its programmed value. It analyzes the deviation and calculates how much heating/cooling energy is required in the process to achieve the programmed value.

Upon completion of the calculation, the controller generates a signal that has the effect of eliminating the error and commands the energy required for the process at the prescribed temperature value by resuming the process of closing and opening the heating element.

In our setup, all electrodonts included fully anatomical mandibular and maxillary teeth with orthodontic brackets featuring 0.022-inch slots (GC Axcess Roth). Two types of wax were used: a pink base layer (Base plate wax; Regular; Kerr Corporation, Brea, CA, USA) and a sticky top layer (Sticky wax; Kerr Corporation). The electrodonts were constructed by positioning the teeth with clinical roots in a silicone mold filled with dual consistency wax, following the user protocol. Each tooth root was wrapped in a copper coil, and a continuous electric current was applied, heating the wax and inducing orthodontic movement through the action of the orthodontic wires.

The bracket system used was made with GC slot 0.22, all TiNb wires (Morita, Fukui, Japan), and NiTi and SS wires (GC Orthodontics, Tokyo, Japan). We ligated all brackets using SS wire ligatures 0.008 for a more precise position of the wire in the bracket slot, and for posterior anchorage, we used a transpalatal arch. We have started orthodontic treatment on all electrodonts’ arches, and as a measure of success, we have followed the repositioning of upper ectopic canines and space closure in order to obtain an equilibrated maxillary arch.

Every upper arch bracket was ligated, and injection of heat into the resistive material that wraps the roots was programmed for 60 min for each wire size. After assessing the results, the orthodontist decides to proceed to ligate the next wire in the bracket slots, if the levelling and aligning could permit, within safe limits of force delivered to the dental units. After each change of orthodontic wires, we scanned the upper arch using Medit i600 (Medit, Seoul, Republic of Korea). On the scan models obtained after each stage, we start measuring the Little Irregularity index using the Medit measuring tool. All scans and measurements were performed by a single orthodontist. In the end, we studied five TiNb C, ten TiNb R1, ten TiNb R2, fifteen NiTi C wires, five NiTi R1, five NiTi R2, and five SS R1 wires using a stereo microscope to find indentation and metal deformation during repositioning of the ectopic canines and closure of spaces. The codification of the studied arch wires is described in Table 1.

Our initial protocol contained two study groups:

- NiTi group—I.
- TiNb group—II.

For the NiTi group, the protocol selected for this study contains three sizes of orthodontic wires: NiTi C1, R1, and R2 for the levelling and aligning stage and SS R1 stainless steel wire for the finishing stage.

For the TiNb group, the protocol selected for this study contains the same three sizes of orthodontic wires: TiNb C, R1, R2, for the levelling and aligning stage, eliminating SS R1 stainless steel needed for the finishing stage. Deformation of TiNb C orthodontic wires made us change the initial protocol and use them for an additional 40 min a NiTi wire in order to perform the initial alignment and levelling.

The obtained results made us create a third study group where we eliminated the TiNb C.

The experiment design related to arch wire structure is described in Table 2.

Study group I consisted of 36 maxillary arches in which the NiTi C1 orthodontic wires were ligated with 0.008 steel ligatures. These were introduced into the orthodontic simulator, setting the working temperature at 24 degrees Celsius in the calorimetric chamber, the monitoring temperature of the arch at 24 degrees Celsius and the exposure time to the current injection of 60 min divided into ten-minute pulses, with the possibility of evaluating the leveling degree. In the literature, the estimated time to treat an orthodontic case with ectopic canines and premolar extractions varies between 18 and 30 months [17].

The OSS can simulate in ten minutes an in vitro exposure equivalent to 1 month of treatment in vivo, and, as movement, we correlate 1 mm for 10 min, so the exposure of 60 min can emulate 6 months of treatment. The OSS experiments were under direct observation by the operator. The alignment of the ectopic canine bracket slot, in the plane made by the central and lateral incisors, the premolar II and the molar I bilaterally, is monitored, so that the transition to the next NiTi R1 arch in the treatment sequence can be achieved. After bringing the ectopic canine slot into plane, we could move on to the NiTi R1 arch wire, which is ligated with 0.008 steel wire and reinserted into the simulator.

To complete the alignment phase, the NiTiR2 arch wire will be used, also using a metal ligature for brackets. Space closure, after leveling and aligning the dental units, will be performed on the SS arch wire using an elastic chain.

Study group II was composed of 36 maxillary arches with the same variables in vertical and transversal planes reported to the occlusal plane, in which the TiNb C1 orthodontic wires were ligated with 0.008 steel ligatures. These were introduced into the orthodontic simulator, the working temperature set to 24 degrees Celsius at the enclosure level, the arch monitoring temperature to 24 degrees Celsius and the exposure time to current injection to 60 min in 10-min pulses, in which the degree of leveling is evaluated.

## 3. Results

At the beginning of treatment, the canine ectopic positions selected for treatment according to the vertical and transversal planes, for all 36 cases, led to variations in treatment time. The following table shows the times required to perform the simulated orthodontic treatment. As can be seen, there were cases that could not be aligned due to the change in shape of the titanium niobium orthodontic wires.

In cases with a vertical deviation of 1 or 2 mm, we observed that we can perform orthodontic treatment through any of the three proposed protocols, if the transversal deviation is less than 3 mm. The treatment period is 60 min shorter for groups two and three.

If the transverse deviation is 4 mm or greater, an irreversible change occurs in the TiNb wire, which no longer allows the orthodontic treatment to continue.

In cases with a vertical deviation of 3 mm, the irreversible modification of the wire used in the second batch already occurs from a transverse deviation of 3 mm.

In cases where the vertical deviation is at least 4 mm, the wires in the second group suffer this irreversible change, no matter how small the transverse deviation is.

Based on this information, we considered it useful to quantify the deviation as the sum of vertical and transverse deviations. We were thus able to observe the existence of a borderline at a sum of 5 mm and below, for which cases can be finalized with any of the three groups, except in the case where the vertical deviation is 4 mm, when even if we have a transverse deviation of 1 mm, the blockage occurs anyway. Thus, after differences greater than 4 mm in the transverse, vertical, or combined planes, the wire suffers a permanent deformation, the ectopic canine begins to descend in the early stages of treatment and stops at a high position, and treatment cannot be continued.

The total time diagram for the study groups regarding the severity of canine ectopic position in vertical and transverse planes is described in Table 3.

We, therefore, considered the sum of the transverse deviation and the vertical deviation as a severity factor of the orthodontic anomaly. Depending on its value, we divided the cases into cases with reduced severity with a severity value below 5, medium severity with a value between 5 and 8, or high severity with a value over 8. For situations in groups two and three, we have a reduction in orthodontic treatment time by 60 min.

**Figure 2:**
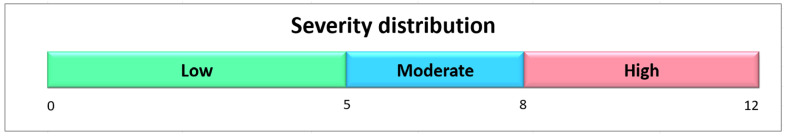
Severity distribution by the canine ectopy reflected in treatment time.

We performed a statistical analysis of the times required for treatment, depending on the severity of the anomaly, and observed that the reduction in the treatment period becomes less and less significant as the severity increases, as it is shown in Figure 2.

A Kruskal–Wallis test was conducted to determine if there were differences in the percentual variation between groups that differed in their severity level: Low (n = 10), Medium (n = 16), and High (n = 10). Distributions of variation were similar for all groups, as assessed by visual inspection of a boxplot. Median variations were statistically significantly different between the different levels of severity, χ^2^(2) = 30.868, *p* < 0.0005. Subsequently, pairwise comparisons were performed using Dunn’s procedure with a Bonferroni correction for multiple comparisons, with statistical significance accepted at the *p* < 0.0166 level. This post hoc analysis revealed statistically significant differences in median variations between all combinations of groups.

The severity level distribution based on the Kruskal-Wallis test is described in Table 4.

**Figure 3:**
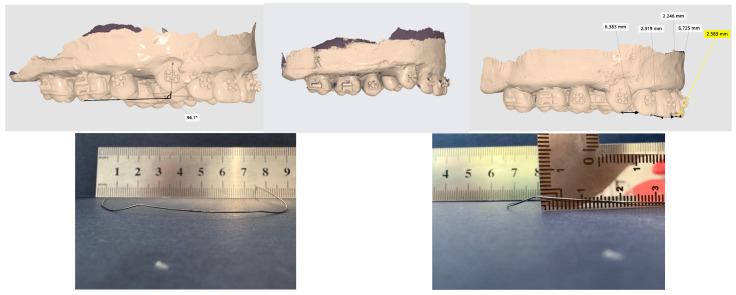
Permanent deformation of TiNb 0.016 archwire observed in the vertical and transversal planes, after removing the archwire; highest amount of deformation is at level of canine bracket slots.

On 28 TiNb C wires, we found an irreversible change in form, remaining deformed after performing the levelling and aligning. This change can be explained by the diagram of hysteresis and frictional forces, and by the way TiNb absorbs and discharges the force to dental units. While the wire remained modified, the orthodontic treatment stopped, leaving the canine in a higher position in vertical, transversal, or combined planes, so we could not proceed to the next TiNb R1 arch. The level of deflexion was different and related to case severity, linked to canine position. Having high angles of the wire in the slot of the bracket and associated with the distance between canine, lateral incisor and second bicuspid will lead to higher deformation of TiNb C orthodontic archwires, as shown in Figure 3.

All rectangular archwires NiTi R1, R2; TiNb R1, R2; SS R1 did not show any permanent form modification.

Using simulated orthodontic treatment in vitro and observing how the treatment of severe class II malocclusions was concluded, made us aware of the necessity of optimizing the use of different orthodontic wires in the stages of aligning, levelling and finishing. Replacing the TiNb C with NiTi C and eliminating the SS wire made the orthodontic treatment time shorter.

**Figure 4:**
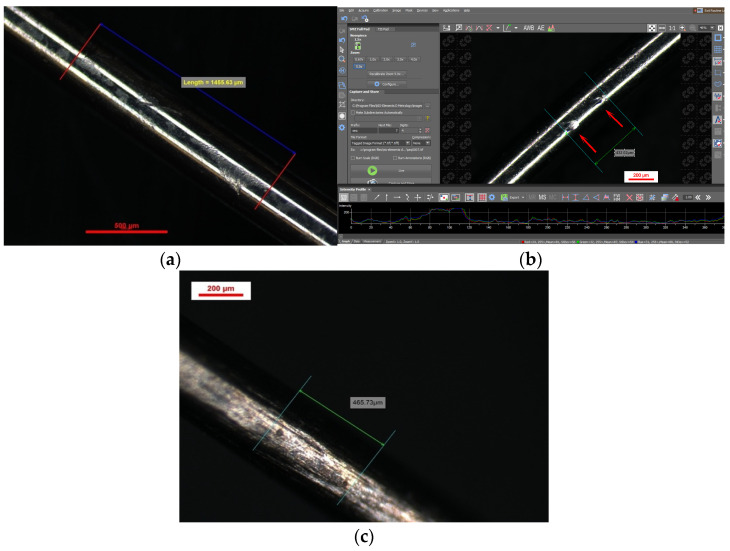
Indentations spectroscopy for 0.016 NiTi (**a**,**b**) and 0.016 TiNb (**c**).

We found a significant number of indentations on TiNb vs. SS, and this comes in support of the theory regarding resistance to sliding of TiNb, as shown in Figure 4.

**Figure 5:**
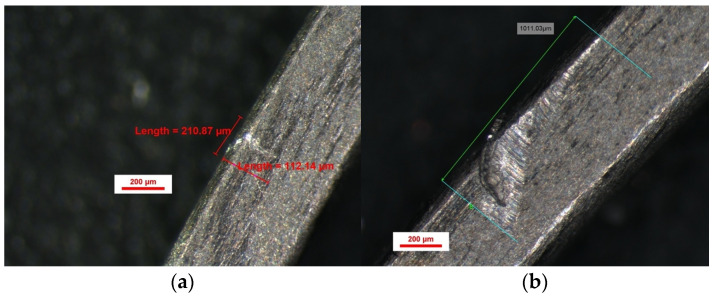
Indentations spectroscopy for 0.016 × 0.022 TiNb (**a**,**b**).

**Figure 6:**
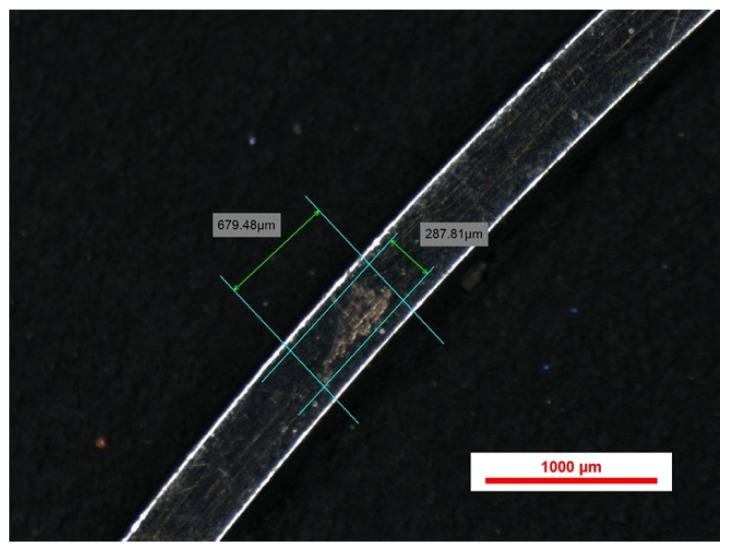
Indentations spectroscopy for 0.019 × 0.025 TiNb.

The indentation pattern for NiTi was gap-like in the material; instead, the TiNb indentation pattern was more scratch-like, as shown in Figure 5 and Figure 6.

## 4. Discussion

Clinical experience has shown us that the duration of orthodontic treatment varies depending on the severity of the ectopy of the canine position in relation to the vertical and transverse planes [18]. Therefore, in our study, in order to quantify the severity of malpositions in both planes up to 6 mm, we chose to evaluate them millimeter by millimeter. By combining the 6 options for each plane, the 36 clinical situations that we analyzed resulted. The validation of the methodology in our study was obtained through the results of the completion of the simulated treatment and the correlation with the duration of in vivo treatment associated with this type of malocclusion.

It is a common benchmark in orthodontics that average tooth movement is 1 mm/month [19]. On our OSS, a movement of 1 mm is achieved in 10 min; therefore, we consider that ten minutes in OSS is equivalent to one month in the oral cavity.

Forces needed to overcome friction in vitro are higher than those in vivo, as claimed by Ho and West [20]. Lubrication plays a role in reducing frictional forces between brackets and wires during in vitro tests with different types of human and artificial saliva [21]. This fact may be a limitation of the present in vitro study conducted in dry conditions. Fortunately, in vitro studies of archwires conducted in dry conditions present rankings of frictional forces and order of frictional values similar to wet conditions and can provide orthodontists with valuable and relevant clinical information [21].

On the other hand, it is difficult to be completely certain how precisely laboratory equipment could recreate the same in vivo situation with the response of the periodontal ligament to orthodontic forces [22]. At the moment, tooth movement cannot be completely imitated in vitro [23,24]. Permanent deformation of TiNb wires led us to change the protocol. We did not find any reference in the literature about permanent deformation of TiNb wires related to high canine position and severe malocclusion treated with first premolar extraction.

From our simulations when treating various cases of ectopic canines, we propose to begin leveling and aligning with an ultra-elastic wire. In our case, NiTi was used because of the limitations related to the temperature interval, but we can also choose a NiTi wire with thermal memory.

We also did not observe any changes during the closing spaces stage using SS versus TiNb. Changing the protocol improved by 20–40%, given the severity of the case, total time spent in simulation, and this may be translated into less time when treating orthodontic patients with fixed appliances. 20–40% less time for fixed orthodontic patients means fewer incidents related to bracket debonding, fewer lesions caused by brackets, and overall increases the quality of life. If we are addressing patients with severe malocclusions and ectopic canines who are allergic or develop allergies to Nichel, the case must be carefully evaluated before starting or when the allergic event appears. The alternative solution proposed by our team is to treat the case with aligners; the position of the canine will dictate the number of aligners, special attachments and elastics: a high position of the ectopic canine will be reflected in a high cost of treatment.

One of the aims of modern orthodontics is the optimization of treatment, which includes the use of AI applications [25], new devices such as 3D photogrammetry [26], and new materials. Given the evolving landscape of orthodontic materials, TiNb archwires represent a promising development, but their full potential has yet to be realized. Future studies should focus on improving the mechanical properties of TiNb archwires and on exploring heat-activated variants or alloy modifications to enhance their resilience during the early stages of treatment. Additionally, investigating their clinical performance in a controlled patient setting will be essential to validating the findings from this in vitro study.

For patients with severe malocclusions and ectopic canines, particularly those who are allergic or develop sensitivities to nickel, careful case selection remains crucial. In cases where TiNb archwires may not provide optimal results, alternative approaches such as clear aligners with specialized attachments and elastics could be considered. While aligners offer a potential solution, the complexity of certain malocclusions, particularly those involving high-positioned ectopic canines, may necessitate a hybrid approach that integrates different treatment modalities.

Thus, while TiNb archwires offer a promising alternative to NiTi in certain orthodontic applications, their mechanical limitations in early treatment stages must be addressed before they can fully replace traditional archwires. By refining treatment protocols and further investigating their long-term clinical performance, TiNb archwires may play a valuable role in modern orthodontics, particularly for patients requiring nickel-free solutions.

The strength of this study is given by the use of a modified OSS dedicated to orthodontic simulations that allowed mm-by-mm changes in the transverse and vertical planes of parameters related to canine ectopia.

The limitations of the study consist of the fact that this research was conducted in lab conditions; in real life, there are a lot of other factors that can interfere with the final result. Therefore, a possible generalization of this result should be carefully treated.

For example, while working in a controlled environment conducting an in vitro experiment, we could not simulate two important roles of saliva: lubrication and heat transfer. These two roles have much to say about increasing frictional forces in pathologic cases of ion excess in saliva created by oxides, deposits of tartar, or about reducing frictional forces between brackets and wires and reducing or accentuating heat transfer to orthodontic archwires.

As future directions, the OSS used to treat class II severe malocclusions with premolar extraction and ectopic canines will be upgraded to perform the saliva cycle under controlled parameters. The archwires used in this experiment will be further analyzed to determine the loss of weight during the experiment in a dry environment in comparison with archwires in normal conditions of lubrification. Also, the archwires selection for in vitro study will be enlarged, including CuNiTi, nanocoated, new bactericide orthodontic archwires and used for simulated orthodontic treatment.

## 5. Conclusions

This study provides an in-depth comparative analysis of TiNb and NiTi orthodontic archwires for the treatment of Class II severe malocclusions with premolar extractions, utilizing a modified orthodontic scientific simulator. Our findings revealed that while TiNb archwires present a viable alternative to conventional NiTi archwires, they exhibit significant limitations in the initial phases of treatment. The most notable drawback was the permanent deformation observed in the TiNb C archwires during the levelling and aligning stage, leading to compromised tooth movement and treatment delays. This highlights the necessity of modifying clinical protocols when incorporating TiNb archwires, specifically by replacing TiNb C archwires with NiTi to ensure consistent and predictable orthodontic forces.

Through this optimization, we achieved a 20–40% reduction in overall treatment time, which has important clinical implications. Shorter treatment durations are beneficial in reducing the risk of bracket debonding, minimizing soft tissue irritation, and enhancing patient compliance. Additionally, the reduced need for stainless steel finishing wires in the TiNb group suggests that these wires could be a step toward more streamlined orthodontic mechanics, particularly for patients requiring nickel-free alternatives due to hypersensitivity or allergy concerns.

Another critical aspect of this study was the role of friction and deformation patterns. Our stereomicroscopic evaluation revealed that TiNb archwires exhibited scratch-like indentation patterns, in contrast to the gap-like indentations observed in NiTi archwires. These findings suggest differences in frictional resistance, which could influence the efficiency of space closure and overall treatment mechanics. Future research should further investigate these properties, particularly in vivo, to determine how the observed deformations and frictional characteristics translate into real-world orthodontic treatments.

## Figures and Tables

**Table 1:** Codification of used arch wires, by geometrical section and dimension.

Wire	Geometrical Section	Dimension [Inch]	Code
NiTi	Circular	0.016	NiTi c
	Rectangular	0.016 × 0.022	NiTi r1
	Rectangular	0.019 × 0.025	NiTi r2
SS	Rectangular	0.019 × 0.025	SS r1
TiNb	Circular	0.016	TiNb c
	Rectangular	0.016 × 0.022	TiNb r1
	Rectangular	0.019 × 0.025	TiNb r2

**Table 2:** Experiment design related to arch wire structure.

Nr Crt	Exp Type	Stages/Wires			
		Levelling	Aligning		Finishing
I	NiTi	NiTi C	NiTi R1	NiTi R2	SS
II	TiNb	TiNb C	TiNb R1		TiNb R2
III	Experiment Solution for Severe Malocclusion with Ectopic canines	NiTi C	TiNb R1		TiNb R2

**Table 3:** Total time diagram for all study groups regarding the severity of canine ectopic position in vertical and transverse planes.

	GROUP I					GROUP II				GROUP III			
	NiTiC	NiTiR1	NiTiR2	SS	TT G I	TiNbC	TiNbR1	TiNbR2	TT G II	NiTiC	TiNbR1	TiNbR2	TT G III
CASE 1.1	10	40	40	60	150	10	40	40	90	10	40	40	90
V: 1 mm													
T: 1 mm													
CASE 1.2	20	40	40	60	160	20	40	40	100	20	40	40	100
V: 1 mm													
T: 2 mm													
CASE 1.3	30	40	40	60	170	30	40	40	110	30	40	40	110
V: 1 mm													
T: 3 mm													
CASE 1.4	40	40	40	60	180	40	-	-	NA	40	40	40	120
V: 1 mm													
T: 4 mm													
CASE 1.5	50	40	40	60	190	40	-	-	NA	50	40	40	130
V: 1 mm													
T: 5 mm													
CASE 1.6	60	40	40	60	200	40	-	-	NA	60	40	40	140
V: 1 mm													
T: 6 mm													
CASE 2.1	20	40	40	60	160	20	40	40	100	20	40	40	100
V: 2 mm													
T: 1 mm													
CASE 2.2	30	40	40	60	170	30	40	40	110	30	40	40	110
V: 2 mm													
T: 2 mm													
CASE 2.3	40	40	40	60	180	40	40	40	120	40	40	40	120
V: 2 mm													
T:3 mm													
CASE 2.4	50	40	40	60	190	40	-	-	NA	50	40	40	130
V: 2 mm													
T: 4 mm													
CASE 2.5	60	40	40	60	200	40	-	-	NA	60	40	40	140
V: 2 mm													
T: 5 mm													
CASE 2.6	70	40	40	60	210	40	-	-		70	40	40	150
V: 2 mm													
T: 6 mm													
CASE 3.1	30	40	40	60	170	40	40	40	120	30	40	40	110
V: 3 mm													
T: 1 mm													
CASE 3.2	40	40	40	60	180	40	40	40	120	40	40	40	120
V: 3 mm													
T: 2 mm													
CASE 3.3	50	40	40	60	190	40	-	-	NA	50	40	40	130
V: 3 mm													
T: 3 mm													
CASE 3.4	60	40	40	60	200	40	-	-	NA	60	40	40	140
V: 3 mm													
T:4 mm													
CASE 3.5	70	40	40	60	210	40	-	-	NA	70	40	40	150
V: 3 mm													
T: 5 mm													
CASE 3.6	80	40	40	60	220	40	-	-	NA	80	40	40	160
V: 3 mm													
T: 6 mm													
CASE 4.1	40	40	40	60	180	40	-	-	NA	40	40	40	120
V: 4 mm													
T: 1 mm													
CASE 4.2	50	40	40	60	190	40	-	-	NA	50	40	40	130
V: 4 mm													
T: 2 mm													
CASE 4.3	60	40	40	60	200	40	-	-	NA	60	40	40	140
V: 4 mm													
T: 3 mm													
CASE 4.4	70	40	40	60	210	40	-	-	NA	70	40	40	150
V: 1 mm													
T: 4 mm													
CASE 4.5	80	40	40	60	220	40	-	-	NA	80	40	40	160
V: 1 mm													
T: 5 mm													
CASE 4.6	90	40	40	60	230	40	-	-	NA	90	40	40	170
V: 1 mm													
T: 6 mm													
CASE 5.1	50	40	40	60	190	40	-	-	NA	50	40	40	130
V: 5 mm													
T: 1 mm													
CASE 5.2	60	40	40	60	200	40	-	-	NA	60	40	40	140
V: 5 mm													
T: 2 mm													
CASE 5.3	70	40	40	60	210	40	-	-	NA	70	40	40	150
V: 5 mm													
T: 3 mm													
CASE 5.4	80	40	40	60	220	40	-	-	NA	80	40	40	160
V: 1 mm													
T: 4 mm													
CASE 5.5	90	40	40	60	230	40	-	-	NA	90	40	40	170
V: 1 mm													
T: 5 mm													
CASE 5.6	100	40	40	60	240	40	-	-	NA	100	40	40	180
V: 1 mm													
T: 6 mm													
CASE 6.1	60	40	40	60	200	40	-	-	NA	60	40	40	140
V: 6 mm													
T: 1 mm													
CASE 6.2	70	40	40	60	210	40	-	-	NA	70	40	40	150
V: 6 mm													
T: 2 mm													
CASE 6.3	80	40	40	60	220	40	-	-	NA	80	40	40	160
V: 6 mm													
T: 3 mm													
CASE 6.4	90	40	40	60	230	40	-	-	NA	90	40	40	170
V: 1 mm													
T: 4 mm													
CASE 6.5	100	40	40	60	240	40	-	-	NA	100	40	40	180
V: 1 mm													
T: 5 mm													
CASE 6.6	110	40	40	60	250	40	-	-	NA	110	40	40	190
V: 1 mm													
T: 6 mm													

**Table 4:** Severity level distribution based on the Kruskal–Wallis test.

Severity Level	Median Percentual Decrease	Overall *p* *	Group Comparisons (*p* **)		
			Low	Moderate	High
Low	35.29%		-	0.006 ^#^	<0.0005 ^#^
Moderate	30.00%	<0.0005	-	-	0.006 ^#^
High	26.08%		-	-	-

* Kruskal-Wallis H test. ** Post-hoc analysis, adjusted significance. ^#^ Significant *p*-value.

## Data Availability

The authors declare that the data from this research are available from the corresponding author upon reasonable request.
